# Investigating the antimicrobial activity, cytotoxicity, and action mechanism of acylated and amidated derivatives of AurH1 antifungal peptide

**DOI:** 10.1186/s12866-023-03090-7

**Published:** 2023-11-09

**Authors:** Reyhane Nikookar Golestani, Elahe Ghods, Mosayeb Rostamian, Hamid Madanchi, Ahmad Farhad Talebi

**Affiliations:** 1 Department of Microbial Biotechnology, Faculty of New Sciences and Technologies, Semnan University, Semnan, 35131-19111 Iran; 2 Department of Biotechnology, School of Medicine, Semnan University of Medical Sciences, Semnan, 35131-38111 Iran; 3 Infectious Diseases Research Center, Health Institute, Kermanshah University of Medical Sciences, Kermanshah, Iran; 4 Nervous System Stem Cells Research Center, Semnan University of Medical Sciences, Semnan, Iran; 5 Drug Design and Bioinformatics Unit, Medical Biotechnology Department, Biotechnology Research Center, Pasteur Institute of Iran, Tehran, 13198 Iran

**Keywords:** Antimicrobial peptides, Aurein1.2, AurH1, Acylation, Amidation, Antifungal peptides

## Abstract

**Background:**

The increasing growth of microbial resistance threatens the health of human societies. Therefore, the discovery and design of new antibiotics seem necessary. Today, antimicrobial peptides (AMPs) are receiving attention due to their unique properties. In our previous studies, exclusive antifungal effects of AurH1, which is a truncated and modified form of Aurein1.2, were synthesized. In this study, AurH1 antifungal peptide was synthesized into acylated (Ac-AurH1) and amidated (AurH1-NH_2_) derivatives, and their antifungal activity, cytotoxicity, anticancer activity, hemolytic effects were investigated. **Finally, the time- of killing, the action mechanism of amidated and acylated peptides, and the effects of salts and human serum on their antimicrobial potency were determined.** All the results obtained about these peptides were compared with the AurH1 without chemical modifications.

**Results:**

The results showed that amidation at the C-terminal of AurH1 compared to acylation at the N-terminal of it can improve the antifungal properties and cytotoxicity of AurH1. **The results showed that AurH1 amidation can maintain the antifungal activity of this peptide in the culture medium containing specific dilutions of human serum compared to the intact AurH1**. Also, the amidation of the C-terminal of AurH1 could not affect the mechanism of action and its time -of killing.

**Conclusion:**

As a result, the amidation of the C-terminal of the AurH1 is a suitable strategy to improve **its antifungal properties and cytotoxicity.** This modification can enhance its properties for animal studies.

## Introduction

In this century, the emergence and spread of antimicrobial resistance (AMR) is a severe threat to public health. This problem is related to the inappropriate and widespread use of common antibiotics in human and veterinary medicine [1, 2]. The increase in the prevalence of opportunistic fungal infections in the community and hospitals in recent years is very worrying because it causes high mortality, especially in patients with primary and acquired immunodeficiency such as HIV/AIDS, autoimmune patients, cancer patients, or transplant recipients [3]. For example, after the rapid spread of COVID-19 in 2019, the rate of fatal fungal infections called Mucormycosis, popularly known as black fungus, increased significantly in the community due to the weakening of the immune system in patients with COVID-19 [4]. However, the use of many existing antifungal compounds is limited due to high toxicity and side effects, ineffectiveness, and the prevalence of strains resistant to these antifungal compounds discovery and design of new antifungal drugs seem very necessary [5].

AMPs are molecules produced by the innate immune system of living organisms, and due to their promising properties, they are considered a suitable alternative to existing antifungal compounds [6]. AMPs show a wide range of antimicrobial activities, including antibacterial, antifungal, antiviral, and anticancer, and also overcome microbial drug resistance. Other biological functions of AMPs include apoptosis induction, wound healing, and immune system modulation [7, 8]. Antifungal peptides (AFPs) are a subgroup of AMPs, most of which are amphipathic and lead to cell lysis by binding to the membrane of fungi. Other AFPs lead to pore formation and leakage of intracellular contents by interacting with the cell wall components [9]. Also, some AFPs kill fungi by inhibiting the synthesis of some essential compounds of fungal cells, such as glucan and chitin molecules [9]. Aurein is a group of cationic peptides derived from the secretions of skin glands of two Australian frogs named *Litoria aurea* or *Litoria raniformis* [6, 10]. These cationic peptides have a high affinity to the anionic membranes, including the membrane of cancer cells or microbial cell walls. Usually, this peptide interacts with the membrane on the surface and disturbs the integrity of the membrane depending on the concentration [11]. The AurH1 is a new heptapeptide derived from the Aurein1.2, designed in 2019 by Madanchi et al. AurH1 has specific and exclusive fungicidal activity without toxic effects on Gram-positive and Gram-negative bacteria and human cells, while Aurein1.2 has both antifungal and antibacterial activity [6]. The use of natural AMPs faces some challenges and problems like toxicity, instability, inflammation, sensitizing, and high cost of synthesis. Residue alterations and substitution, targeted truncation, and some modifications on these peptides based on computational methods and protein engineering strategies can be a way to overcome these limitations [12, 13]. However, chemical changes in peptides are another method of modifying their biological properties. Post-translational modifications are necessary for the antimicrobial activity of some AMPs. Also, chemical modification of AMPs can improve their properties, such as antimicrobial potency, cytotoxicity, and stability. The most common of these artificial chemical modifications are carboxy-amidation and N- N-terminal acylation. Amidation is a minor modification in which peptides terminate with an amide group (–NH_**2**_) instead of a carboxyl group (–COOH). N-terminal acylation of peptides is the chemical reaction in which an acyl group (R − C = O group such as fatty acid) is added to a primary amine group (NH_**2**_-) of peptides [14].

In this study, AurH1 antifungal peptide from our previous research (AurH1) was synthesized into acylated and amidated forms. Next, the antimicrobial activity of these chemical-modified AurH1 and their cytotoxicity on human skin fibroblast cells, two selected cancer cell lines, and red blood cells were investigated. Finally, the action mechanism of amidated and acylated forms of AurH1 was evaluated. All the results obtained about these peptides were compared with the AurH1 without chemical modifications.

## Materials and methods

### Peptide synthesis

The acylated and amidated forms of AurH1 peptides with WFKIIKK sequence [6] were synthesized using the fluorenyl methoxycarbonyl (Fmoc) method along with the acyl (Ac) chain at the N-terminal and amine (NH_**2**_) group at the C-terminal of the peptide by PepMic Co Ltd (Jiangsu Sheng, China). Peptides were purified to 95% purity using C18 reverse-phase high-performance liquid chromatography (RP-HPLC). The molecular weight of the synthetic peptides was determined by mass spectrometry analysis on a Sciex API100 LC/MS mass spectrometer (Perkin Elm Co., Norwalk, CT, USA) in positive ion mode.

### Reagents and compounds

Sabouraud dextrose agar (SDA), Sabouraud dextrose broth (SDB), Mueller Hinton Broth (MHB), Mueller Hinton agar (MHA), ethanol, phosphate-buffered saline (PBS), penicillin, and streptomycin were purchased from Merck Millipore Company (Merck, Darmstadt, Germany). Fetal bovine serum (FBS) and Roswell Park Memorial Institute (RPMI) 1640 medium and DMEM F12 media were purchased from Gibco Company (Gibco, Carlsbad, CA, USA). Cell culture antibiotics (penicillin, streptomycin, and cyclosporin), nystatin, fluconazole, amphotericin B, trypsin, trypan blue dye, NaOH, HCl, 3‐(4,5‐dimethylthiazol‐2‐yl) ‐2,5‐diphenyltetrazolium bromide (MTT) dye, Triton‐X‐100, glutaraldehyde, and dimethyl sulfoxide (DMSO) were procured from Sigma (Sigma‐Aldrich, St. Louis, MO, USA).

### Cell lines, bacterial and fungal strains

*Staphylococcus aureus* (ATCC 25,923), *Escherichia coli* (ATCC 25,922), *Candida albicans* (ATCC 10,231), *Candida glabrata* (ATCC 90,030), *Candida krusei* (ATCC 28,870), *Aspergillus niger* (ATCC 9142), and *Aspergillus flavus* (ATCC 24,109) were purchased from Microbial bank and Pathogenic Fungi Culture Collection of Iran (Pasture Institute of Iran). The human skin fibroblast (Hu02 cell) and human breast cancer cells (MDA-MB-231 and SKBR-3 cell lines) were purchased from the National Cell Bank of Iran, Pasture Institute of Ira) for cytotoxicity test.

### Antimicrobial assay

Antimicrobial effects of the Ac-AurH1, AurH1-NH_2_, and AurH1 against the selected bacteria and fungi were conducted using a standard serial dilution method in a 96-well microplate. For this purpose, the desired fungal and bacterial strains were grown overnight at 37 °C (Some of the fungi were incubated at 25 °C) in related media (SDA for fungi and MHA for bacteria). Then, a standard microbial inoculum of 0.5 McFarland was prepared in MHB and SDB for bacteria and fungi, respectively. Next, serially diluted synthetic peptides in a volume of 100 µL were added to the microtitre plates, followed by 100 µL of bacteria and fungi to give a final inoculum of 5 × 10^5^ colony-forming units (CFU)/ml (a 0.5 McFarland standard inoculum). Plates were incubated at 37 °C for 24 h, and minimum inhibitory concentrations (MICs) for each peptide were determined. In this test, penicillin and streptomycin antibiotics for bacteria and fluconazole, nystatin, and amphotericin B for fungal strains were used as antibiotic control. To determine the minimum fungicidal concentrations (MFC), 20 µL from the MIC-related well of each peptide and its subsequent wells were cultured on agar media (SDA) and incubated overnight at 37 °C. All tests were repeated three times independently [15].

### Cytotoxicity test

Toxicity of peptides on Human skin fibroblast (Hu02 cells) and two human breast cancer cell lines (MDA-MB-231 and SKBR3 cell lines) measured by an MTT assay. At first, cell lines with a density of 1 × 10^5^ cells were cultured in the wells of 96-well plates for 24 h under standard conditions (37 °C temperature, 5% CO2 atmosphere with 95% relative humidity). After that, the old medium was replaced with fresh medium (with 10% FBS) containing different peptide concentrations, and the cells were incubated for 24 h. A medium without peptide was used as a negative control. After 24 h of incubation and washing the wells with PBS, the medium (containing 10% FBS) was replaced with fresh medium containing 10% MTT solution (freshly prepared 5 mg/ml MTT in PBS), and the plate was incubated for four h in 5% CO2, at 37 °C. Next, the medium was removed from the wells, and 100 µL DMSO was added. Afterward, the plate was shaken to dissolve Formazan crystals [6, 13]. Finally, by a microplate reader (STAT FAX 2100, BioTek, Winooski, USA), the absorbance values were measured at a wavelength of 595 nm. The percentage of toxicity was calculated as follows:

$$Toxicity\% {\text{ }} = \left( {1 - \frac{{mean\,OD\,of\,sample}}{{mean\,OD\,of\,control}}} \right) \times {\text{ }}100$$

$$Viability{\text{ }}\% {\text{ }} = {\text{ }}100{\text{ }} - {\text{ }}Toxicity\%$$

### Hemolytic assay

A hemolytic assay was performed to investigate the effects of peptides on human red blood cells. First, 5 ml of fresh human blood was taken from a volunteer. After that, 20% (v/v) suspension of human erythrocytes was prepared and diluted in PBS (1:20). Next, 100 µL of this blood suspension was added in triplicate to 100 µL of a 2-fold serial dilution series of the peptide in a 96‐well plate. 1% Triton-X 100 was used as a positive control to lyse 100% of RBCs, and sterile 0.9% NaCl solution was used as a negative control. Then the plates were incubated at 37 °C for 1 h and centrifuged at 3500 rpm for 10 min. Next, 100 µL of the supernatant was transferred to a new 96-well plate to measure the absorbance at 414 nm using a microplate reader (STAT FAX 2100, BioTek, Winooski, USA). Finally, the percentage of hemolysis was calculated as follows [16]:

$$\begin{gathered}{\mathbf{Hemolysis}}{\text{ }}\% = \hfill \\\,\,\,\,\,\,\,\,\,\,\,\,\,\,\,\,\,\,\,\,\,\,\,\,\,\,\,\,\left( {\frac{{{\text{Mean}}\,{\text{OD}}\,{\text{of}}\,{\text{sample}} - {\text{Mean}}\,{\text{OD}}\,{\text{of}}\,{\text{negative}}\,{\text{control}}}}{{{\text{Mean}}\,{\text{OD}}\,{\text{of}}\,{\text{positive}}\,{\text{control}} - {\text{Mean}}\,{\text{OD}}\,{\text{of}}\,{\text{negative}}\,{\text{control}}}}} \right) \times 100\% \hfill \\ \end{gathered}$$

### Killing kinetic assay

A killing kinetic assay was conducted against *C. albicans* to determine the fungicidal speed of the peptides. First, logarithmically growing yeasts were inoculated to sterile SDB, adjusted to 5 × 10^5^ CFU/mL, and added to the medium containing the peptides at concentrations equivalent to 1X MFC. Following incubation for 0, 4, 8, 12, and 24 h at 37℃, samples were diluted (1:100) and plated in triplicate onto SDA plates. Then the colonies (Colony forming units or CFUs) were counted. The results were reported based on the logarithm of CFU per specific time points. Also, nystatin and fluconazole antibiotics were used as a control [6].

### Combinatorial effects of peptides with conventional antibiotics

The combinatorial effects of Ac-AurH1 and AurH1-NH_2_ with fluconazole (Flu), nystatin (Nys), and amphotericin B (Amph) against *C. albicans*, *C. glabrata*, and *C. krusei* were investigated by a checkerboard titration method with some of the modifications [6]. The microbial inoculum was prepared based on broth micro-dilution assay to generate a concentration of fungi corresponding to 5 × 10^**5**^ CFU/mL. The tested concentrations of antibiotics and peptides ranged from 1 X MIC to 1/8× MIC, reported following 24 h of non-growth. The obtained results were analyzed in terms of the fractional inhibitory concentration index (FIC):

$$\begin{gathered}{\text{FIC}}\,{\text{index}} = \hfill \\\,\,\,\,\,\,\,\,\,\,\,\,\,\,\,\,\,\frac{{{\text{MIC}}\,{\text{of}}\,{\text{AMP}}\,{\text{in}}\,{\text{combination}}\,{\text{with}}}}{{{\text{MIC}}\,{\text{of}}\,{\text{AMP}}\,{\text{alone}}}} + \frac{{{\text{MIC}}\,{\text{of}}\,{\text{antibiotics in combination with}}}}{{{\text{MIC of antibiotics alone}}}} \hfill \\ \end{gathered}$$

AMPs and antibiotics are antagonistic if FIC ≥ 4.0, indifference if > 1 FIC < 4.0, additive if > 0.5 FIC ≤ 1, and synergism if FIC ≤ 0.5.

### Investigating the effects of the AurH1-NH _2_ peptide on *C. Albicans* surface by FE-SEM

At first, *C. albicans* were cultured in broth media. Next, yeasts were deposited by centrifugation (4000 rpm for 1 min) and washed (Three times) with PBS (pH 7.4). Afterward, a cell suspension with 10^7^CFU was prepared and was incubated with 0.5X MIC concentration of AurH1-NH_2_ peptide for 0, 2, 4, and 6 h and precipitated at 4000 rpm for 1 min. Next, the cells were washed twice with PBS and fixed for 1 h (temperature of 25 °C, dark chamber) with 2.5% glutaraldehyde in 0.1 M PBS [6, 17]. The samples were centrifuged at 4000 rpm for 1 min, washed three times in PBS, and put on the glass slides (1cm2). Subsequently, cells were dehydrated with ethanol gradient (at 10, 30, 60, 70, and 90%). After that, the dehydrated cells were dissolved in 100% ethanol for 15 min and dried at room temperature (25 °C). Finally, *C. albicans* cells were coated with gold nanoparticles by an automatic sputter coater and observed using an FE-SEM instrument (JSM-7610 F, JEOL Co., Japan and MIRA3, TESCAN Co., Czech).

### Ergosterol binding assay

An ergosterol binding assay was performed to measure the binding potency of peptides to fungal membrane sterols. For this purpose, the ergosterol was dissolved in DMSO and Tween-20, it was heated to increase the solubility of the formed emulsion and diluted in an SDB medium at a concentration of 400 mg/ml and incubated at 37 °C for 1 h. After that, MIC of the AurH1, Ac-AurH1, and AurH1-NH_2_ peptides was measured by the micro-dilution method in the presence and absence of ergosterol in different concentrations (1000 to 0.488 mg/ml) against *C. albicans* [17, 18].

### Effect of salts and human serum on the antimicrobial activity of peptides

To evaluate the **effect of salts and human serum on the antimicrobial activity of** the AurH1-NH_2_ and AurH1 peptides, their antimicrobial activity (MICs values) against *C. albicans* under different conditions containing salt and human serum was determined. Therefore, mid-logarithmic phase yeasts diluted to 5 × 10^5^ CFU/ml in SDB were added to related media containing different concentrations of human serum (10%, 20%, and 30%) and salts (150 mM NaCl and 1mM MgCl_2_), and incubated at 37 °C for 24 h [19, 20].

### Statistical analysis

All tests were performed three times. Using a t-test, the SPSS Statistics 22.0 program (SPSS Inc., Chicago, IL, USA) assessed the statistical significance of the variations in the MIC, toxicity, hemolysis, and ergosterol binding activity values of peptides. P values less than 0.05 were regarded as statistically significant.

## Result

### Synthesis and determining the characteristics of chemically modified peptides

**Fig. 1:**
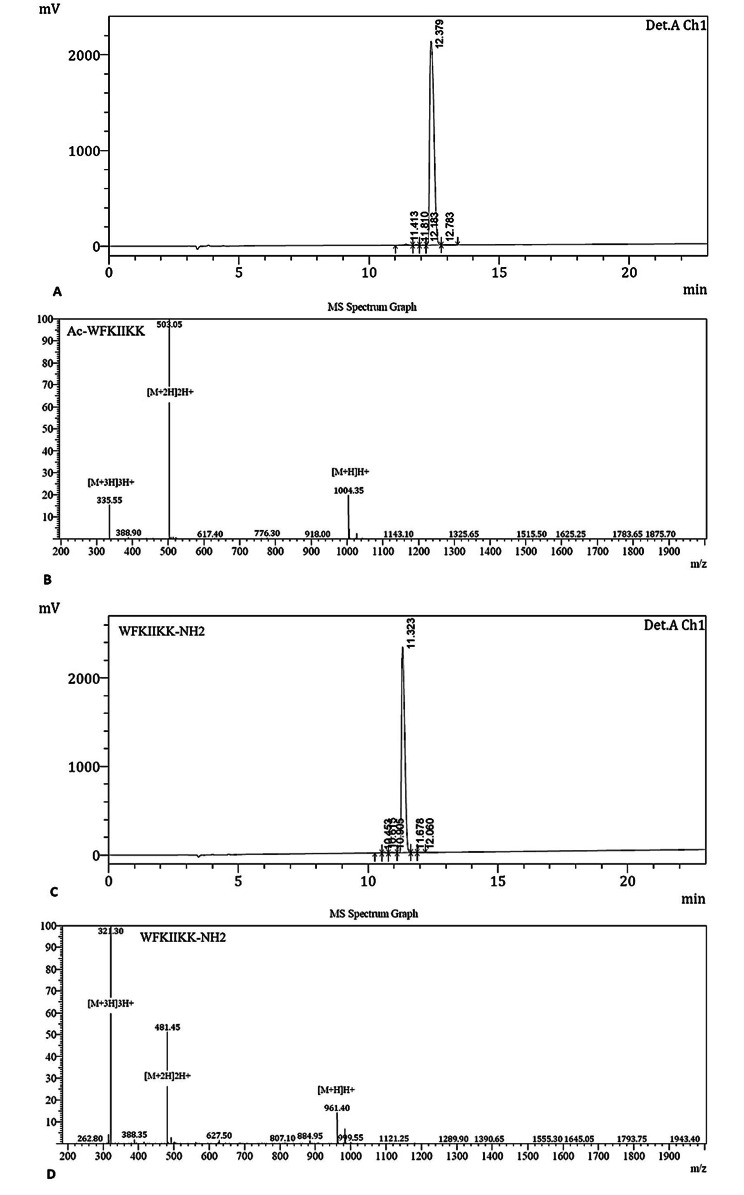
RP-HPLC chromatogram and Mass spectrometry curves for Ac-AurH1 **(A, B)** and AurH1-NH_2_ **(C, D)** confirm the molecular weight and purity of the synthesized peptides. The theoretical molecular weight of Ac-AurH1 and AurH1-NH_2_ is 1004.29 and 961.26 Da, respectively

Acylated and amidated derivatives of AurH1 peptide (Ac-WFKIIKK or Ac-AurH1 and WFKIIKK-NH_2_ or AurH1-NH_2_) were chemically synthesized and purified to > 95%. The quantity and quality of synthetic peptides by RP-HPLC and mass spectroscopy were validated (Fig. 1).

### The inhibitory and killing rate of peptides against microbes

**Fig. 2:**
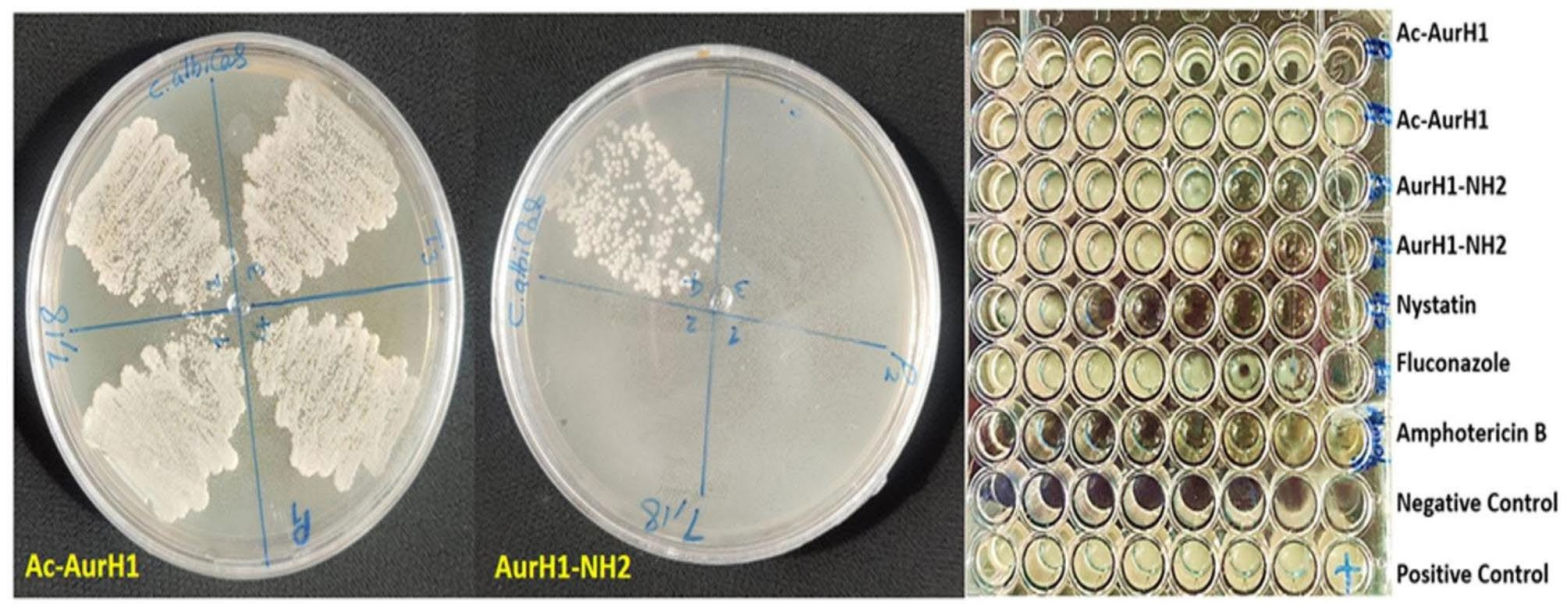
This image shows the MIC assay test microplate and the MFC assay plates against *C. albicans*

The antimicrobial test results showed that Ac-AurH1 and AurH1-NH_2_ peptides, like the AurH1 peptide, did not show any specific antibacterial effects, but the antifungal effects of the AurH1-NH_2_ peptide increased compared to the AurH1. Statistical analysis showed that the fungicidal activity of AurH1-NH_2_ against *C. albicans, C. krusei*, and *C. glabrata* was significantly higher than AurH1 (p < 0.05). However, AurH1-NH_2_ and Ac-AurH1 had no antifungal effect on *A. niger* and *A. flavus*. Based on statistical analysis, Ac-AurH1 showed significantly lower mean MIC values than AurH1-NH_2_ and AurH1 (p < 0.05). In comparison with AurH1-NH_2_, Fluconazole showed fewer antifungal effects against *C. albicans* (Fig. 2) and *C. krusei*. All tests were conducted in triplicate and their mean results are shown in Table 1.

**Table 1:** MICs and MBCs (MFCs) in µg/mL against selected bacteria and fungi

Microorganism	Means of MIC/MBC (MFC) for 3 independent tests						
	**AurH1** ^*^	**Ac-AurH1**	**AurH1-NH**_2_	**Pen**	**Strep**	**Nys**	**Flu**
***S. aureus***	> 1000	> 1000	> 1000	0.45/NA	62.50/NA		
***E. coli***	> 1000	> 1000	> 1000	31.25/NA	3.9/ NA		
***C. abicans***	15.625/125	250/>1000	7.81/62.50			1.95/3.90	62.50/500
***C. glabrata***	125/500	> 1000	62.50/62.50			3.90/3.90	125/500
***C. krusei***	31.25/250	500/>1000	7.81/31.25			1.95/3.90	62.50/250
***A. niger***	125/500	> 1000	125/500			31.25/125	500/>1000
*** A. flavus***	> 1000	> 1000	250/1000			7.81/62.50	> 1000

**Pen**: Penicillin, **Strp**: Streptomycin, **Nys**: Nystatin, **Flu**: Fluconazole

* The results related to AurH1 are included based on the results of our previous study

### MTT assay results

**Fig. 3:**
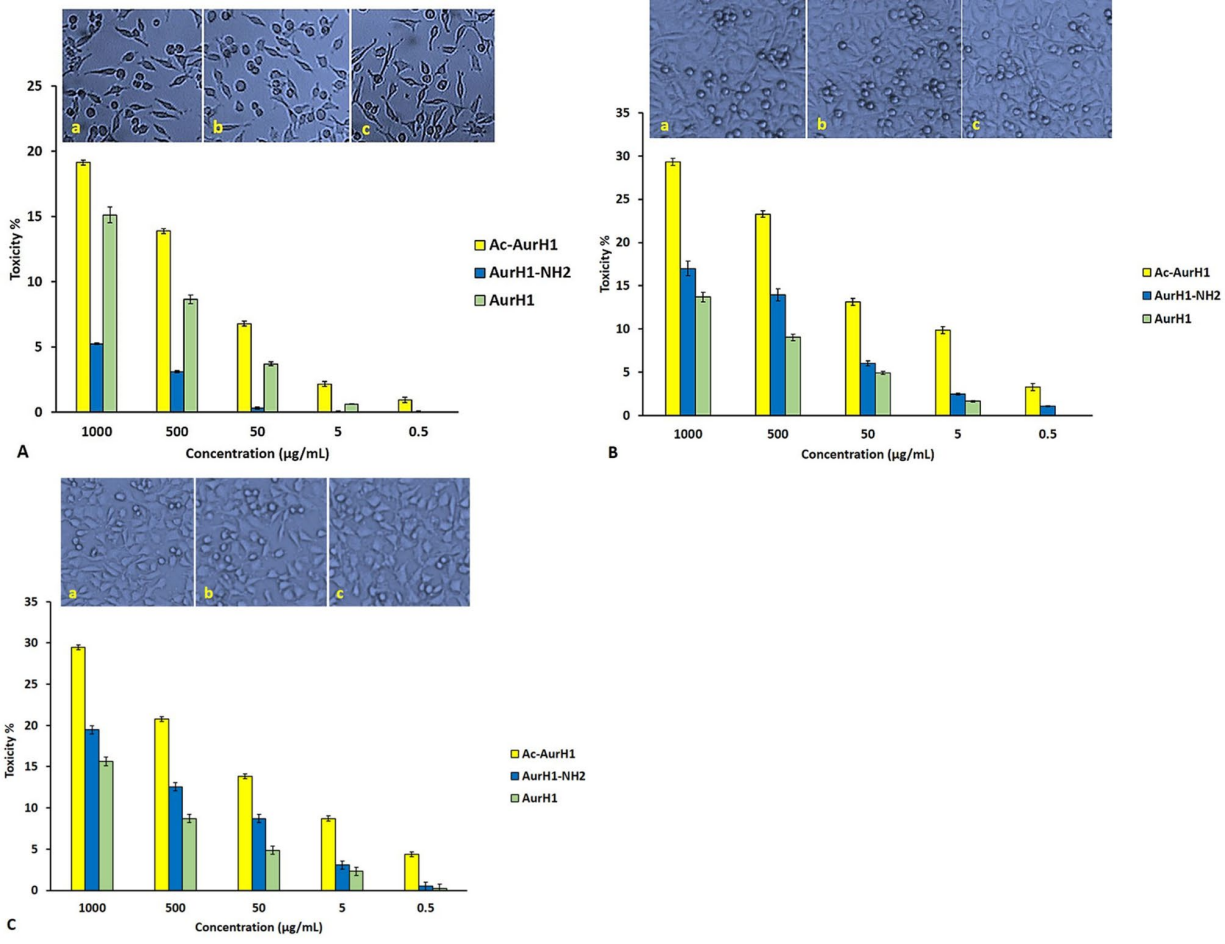
Concentration/toxicity percent chart for peptides against **(A)** Hu02 cell line, **(B)** MDA-MB-231 cells, and **(C)** MCF-7 cell line. In this figure, the letters “**a**” to “**c**” represents the inverted microscope images of the MTT assay microplate wells at a concentration of 1000 for Ac-AurH1, AurH1-NH2, and AurH1, respectively. All of the results are the mean of three independent experiments

The toxicity/concentration charts show the AurH1-NH_2_ peptide has no significant toxicity at its MIC ranges, while the Ac-AurH1 peptide at its MIC ranges had more than 10% toxicity on the Hu02 cells. Also, statistical analysis shows that the toxicity of AurH1-NH_2_ is significantly lower than AurH1(p < 0.05) and Ac-AurH1(p < 0.001) on Hu02 cells. However, the IC_50_ of three peptides on this cell line was > 1000 µg/mL. The toxicity of Ac-AurH1 on MDA-MB231 and MCF-7 was significantly higher than the AurH1 and AurH1-NH_2_ peptides. However, the Ac-AurH1 peptide at its highest concentration (1000 µg/mL) inhibits the growth of less than 30% of breast cancer cells. Unlike Hu02 cells, AurH1-NH_2_ shows more toxicity against the two breast cancer cells (MDA-MB-231 and MCF-7 cell lines) than AurH1 (Fig. 3).

### Hemolysis test results

**Fig. 4:**
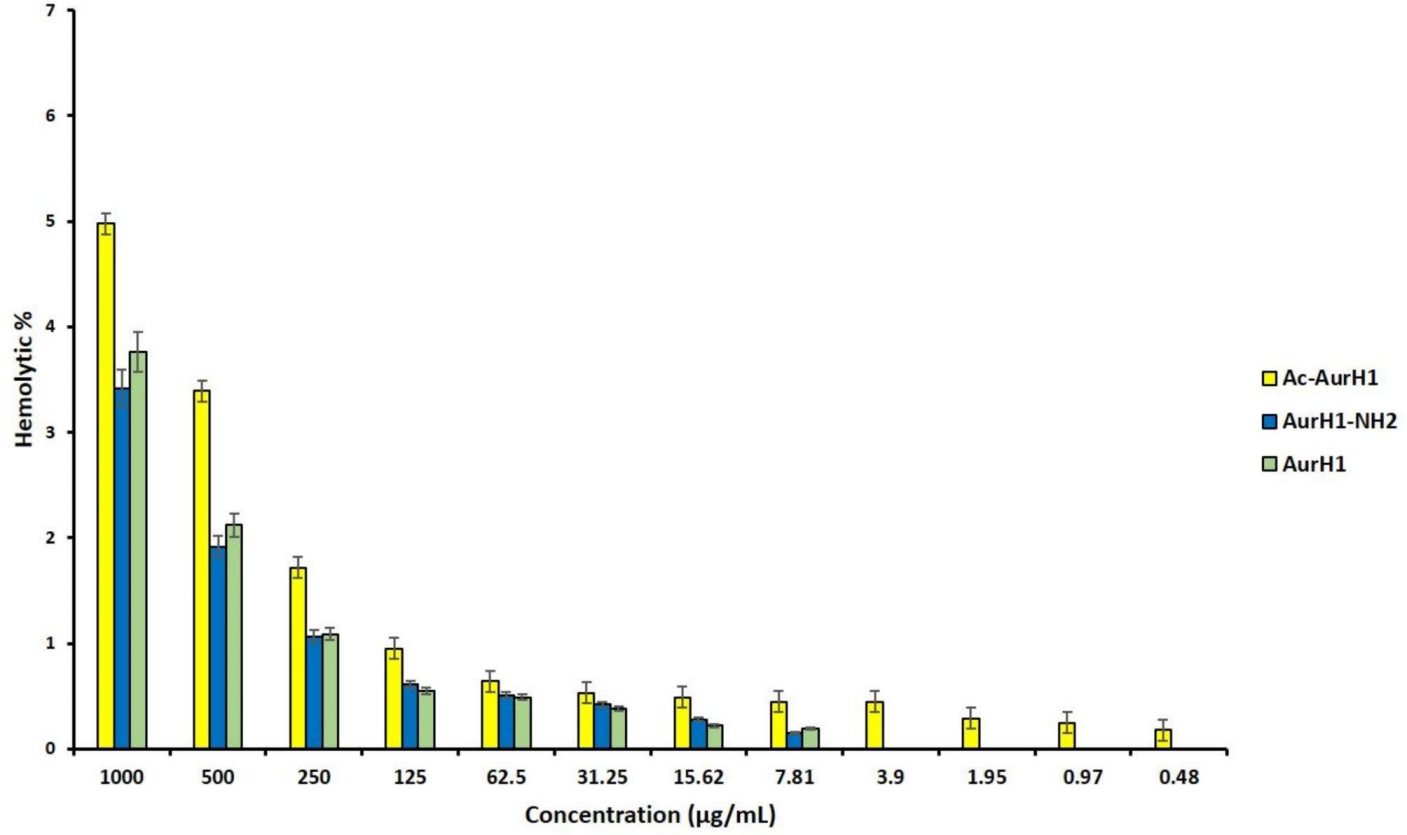
Concentration/ hemolysis percent chart for Ac-AurH1, AurH1-NH_2_, and AurH1 peptides. All of the results are the mean of three independent experiments

The hemolytic activity of all three peptides was less than 5% at their highest concentration (1000 µg/mL). The results showed that the Ac-AurH1 peptide lyses more human red blood cells than AurH1-NH_2_ and AurH1. However, statistical analysis indicated that the hemolysis percentage of all three peptides is not significantly different. All the results are represented as the mean of three independent experiments (Fig. 4). The HC_50_ (a concentration of compounds that lyses 50% of red blood cells) peptides AurH1, Ac-AurH1, and AurH1-NH2 are **13,484, 10,127**, and **14,246** µg/ml, respectively. Therefore, all three peptides do not have any hemolytic activity in their MIC and MFC ranges. As Figure shows, even at the highest concentration (**1000 µg/ml**), all three peptides hemolysis less than 5% of cells.

### Time of killing assay

**Fig. 5:**
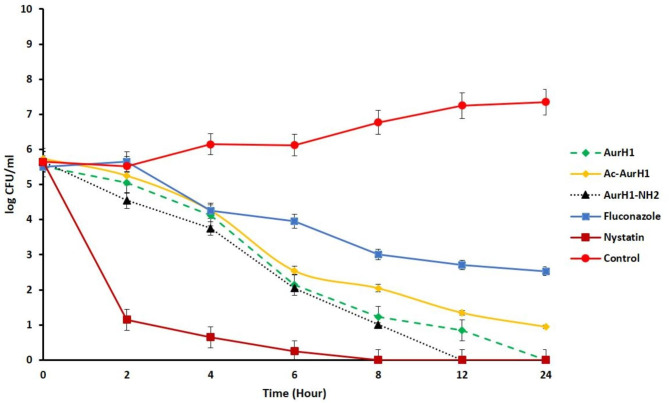
Time of killing curves of Ac-AurH1, AurH1-NH_2_, AurH1, Nystatin, and fluconazole against *C. albicans* at 1X MIC concentration

The time of killing result showed that AurH1-NH_2_ (at 1X MIC concentration) had a maximum fungicidal effect against *C. albicans* after 12 h. This peptide fully killed all the yeasts, while the AurH1 peptide had similar activity after 24 h. Nystatin at 1X MIC concentration could kill all *C. albicans* after 8 h. However, Ac-AurH1 and Fluconazole at the same concentration (1XMIC) could not eliminate *C. albicans* after 24 h (Fig. 5).

### Checkerboard test

The synergism test was performed in laboratory conditions to evaluate the interaction of antibiotic compounds. Based on the FIC index, the test results showed that the simultaneous use of several different antibiotics in treatment could inhibit the pathogenic microorganism with other mechanisms. Synergistic effects between Ac-AurH1 and AurH1-NH_**2**_ peptides with amphotericin B were observed against selected fungi, but no synergistic effect was determined between these two peptides with fluconazole and nystatin antibiotics. Table 2 interprets the results of synergy measurement and the FIC index.

**Table 2:** FIC indexes and their Interpretations for peptides-antibiotics against *C.abicans*, C. *glabrata*, and *C. krusei*

Combination		*C. abicans*		*C. glabrata*		*C. krusei*	
		**FIC index**	**Interpretation**	**FIC index**	**Interpretation**	**FIC index**	**Interpretation**
Ac-AurH1	Flu^a^	0.5	Additive	6	Indifference	2.5	Indifference
Ac-AurH1	Nys^b^	8.2	Antagonist	5.5	Antagonist	10.5	Indifference
Ac-AurH1	Amph^c^	0.489	Synergy	0.123	Synergy	0.06	Synergy
AurH1-NH_2_	Flu^a^	1.25	Indifference	3	Indifference	1.55	Indifference
AurH1-NH_2_	Nys^b^	1.15	Indifference	5.5	Antagonist	7	Antagonist
AurH1-NH_2_	Amph^c^	0.24	Synergy	0.25	Synergy	0.25	Synergy

### Ergosterol assay results

The results of the Ergosterol binding assay showed that Ergosterol does not affect the MIC of peptides because peptides AurH1, Ac-AurH1, and AurH1-NH_2_ do not bind to Ergosterol. Therefore, Ergosterol is not the target for these two peptides. Table 3 shows the results of the Ergosterol binding assay.

**Table 3:** MIC results of AurH1, Ac-Are H1, and Air H1-NH_2_ peptides against *C. albicans* before and after treatment with Ergosterol (Erg)

Treatments	Means of MIC/MBC (MFC) for 3 independent tests
AurH1	15.625
Ac-AurH1	250
AurH1-NH_2_	7.81
Ergosterol (Erg)	> 1000
AurH1/(Erg)	15.625
Ac-AurH1/ (Erg)	250
AurH1-NH_2_/ (Erg)	7.81

### Investigating changes in surface morphology of *C. Albicans* after treatment by AurH1-NH_2_

**Fig. 6:**
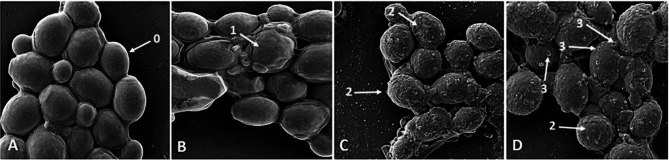
FE-SEM images **(A-D)** indicate *C. albicans* after treatment with 0.5 X MIC of AurH1-NH_2_ at different intervals. *C. albicans***(A)** without peptide treatment, **(B)** with 0.5 X MIC of AurH1-NH_2_ treatment after two hours, **(C)** after four hours, and **(D)** after six hours of treatment. The labels include (0) intact cells, (1) cell surface shrinkage, (2) cell roughening, and (3) cell surface pores

FE-SEM imaging was conducted to evaluate AurH1-NH_2_ peptide effects on *C. albicans* morphology at different intervals. Untreated *C. albicans* by peptide were intact. *C. albicans* after **2 h** of treatment with 0.5 X MIC of AurH1-NH_2_ shrinkages in cell-surface. Following 4 h, an increase was seen in the amount of cell surface shrinkage and cell roughening. The blisters and surface pores on the cell surface were created after 6 h of AurH1-NH_2_ treatment (Fig. 6).

### Effect of human serum and salts on AurH1-NH_2_ peptide antimicrobial activity

The results indicated a significant difference between MIC values of AurH1 and AurH1-NH_2_ in standard SDB and SDB containing 20% and 30% human serum (P < 0.05). However, no significant difference between the antimicrobial activity of the two peptides was seen in the culture medium containing 10% human serum (P > 0.05). The results of the salt’s effect on peptides showed that no significant difference between the antimicrobial effects of the AurH1 and AurH1-NH2 was seen in a medium containing 150 mM NaCl and **1 mM** MgCl2 (Table 4).

**Table 4:** MICs of AurH1 and AurH 1-NH_2_ peptides against *C. albicans* after treatment with salts and different percentages of human serum

Peptides	Means of MIC values against *C. albicans*					
	SDB	SDB + 10% serum	SDB + 20% serum	SDB + 30% serum	SDB + MgCl2	SDB + NaCl
**AurH1**	15.62	15.62	62.5	125	15.62	15.62
**AurH1-NH**_2_	7.81	7.81	7.81	15.62	7.81	7.81

## Discussion

Recently, antimicrobial peptides (AMPs) as a new generation of antibiotics have attracted the attention of many scientists. However, natural AMPs have limitations such as cytotoxicity, immunogenicity, instability in the body, long length, and expensive synthesis [13, 15, 17]. Therefore, modification of AMPs with natural origin can greatly help to overcome these limitations. Modifications can be at the level of sequence and changes in their amino acid content or can be at the level of changes in the type of isomerism of D or L residues. Chemical modification is one of the other important changes to improve the properties of AMPs, which can help to improve properties such as antimicrobial activity and stability of peptides. Truncation of AMPs to the extent that their antimicrobial properties are maintained is a very suitable strategy, because on the one hand, by truncating peptides, their toxicity and immunogenicity are reduced, and on the other hand, this action reduces synthesis costs and increases peptide stability against proteolytic degradation [6, 16].

Aurein peptides are a large family of cationic AMPs secreted from amphibians’ skin as antimicrobial agents from the innate immune system [6, 15, 20]. Many studies have been conducted on the antibacterial properties of the Aurein antimicrobial peptide family, and many studies have identified this family as inhibiting the growth of Gram-positive bacteria [21]. In one of our previous studies in 2019, synthetic derivatives of Aurein1.2 were synthesized (Aurein N1 and Aurein N2) and killed Gram-negative bacteria as well as Gram-positive bacteria [15]. In 2023, we reported the antifungal effects of these two peptides and a newly synthesized derivative named Aurein N3 [20]. In general, there are not many documented reports about the antifungal properties of the Aurein family [20]. In 2019, we designed a 7-amino acid derivative of Aurein 1.2 entitled AurH1 by the truncation strategy and *in silico* tools, which was exclusively antifungal [6]. This peptide exerts most of its antifungal activity on yeasts such as *C. albicans* [6]. Due to the unique antifungal properties and low toxicity of AurH1, in this study, we used the chemical modification strategy of the peptide sequence terminal to improve some of its properties such as toxicity and antifungal potency. Once its end (N-terminal) was acylated and another time its end (C-terminal) was amidated to create two modified derivatives entitled Ac-AurH1 and AurH1-NH_2_.

The results of the antibacterial assay showed that amidated and acylated derivatives of AurH1, like their parent peptide, have no antibacterial activity against Gram-negative and positive bacteria. This issue can be related to the short length of AurH1. Previous studies have also confirmed that truncating antimicrobial peptides can reduce their bactericidal potency and cytotoxicity [6, 22, 23]. Also, amino acids with high frequency in antifungal peptides have been replaced in the AurH1 sequence, which may not cause the antibacterial properties of Aurein1.2 to appear [6]. Instead, antifungal tests indicated that amidation of the C-terminal of AurH1(AurH1-NH_2_) could improve their antifungal properties against yeasts (but not molds). On the contrary, the acylation of AurH1 (Ac-AurH1) at the N-terminal not only did not improve this activity but also caused a decrease in the antifungal properties of the Ac-AurH1.

MTT results on normal human fibroblast cells (Hu02 cell line) showed that amidation at the C-terminal of AurH1 does not play a role in increasing its cytotoxicity. Therefore, the toxicity of AurH1-NH_2_ in this cell line is less than that of the parent peptide (AurH1). On the other hand, acylation at the end of AurH1 (Ac-AurH1) increases the cytotoxicity of this peptide on this cell line compared to the parent peptide. This issue can be related to the increase of hydrophobicity of Ac-AurH1 compared to other peptides. The cytotoxicity results on breast cancer cell lines (MDA-MB-231 and MCF-7) showed that Ac-AurH1 exerted the highest toxicity on these cells. Another interesting result was that against these two cancer cell lines, the AurH1-NH_2_ showed more toxicity than the AurH1.

This result could be related to the more positive charge of AurH1-NH_2_ than the parent peptide, which is more reactive to the cancer cell membrane (Highly negatively charged) [24]. The results of the hemolysis test also confirmed the cytotoxicity results, so the highest hemolytic activity is related to Ac-AurH1 and the lowest was related to the amidated derivative (AurH1-NH_**2**_). Therefore, here, peptide amidation reduced the toxicity of human erythrocytes.

The results of the time of-killing test showed that the chemical modifications at the end of the AurH1 sequence are more effective on the fungicidal potency than killing speed. These results indicated that the acylation in AurH1 caused this peptide to be unable to kill all fungi even after 24 h. However, there is no significant difference in the fungicidal speed of all three peptide derivatives up to 8 h, while at 12 h, AurH1-NH_2_ was able to kill all the *C. albicans*, but AurH1 shows the same effect after 24 h. Therefore, it can be said that amidation in AurH1-NH_2_ has relatively improved its fungicidal speed, which may be related to its additional positive charge compared to the other two peptides, which can help the peptide to react faster with its target [25].

The results of the combined treatment of AurH1-NH_2_ with fluconazole, nystatin, and amphotericin B showed that this peptide only synergizes with amphotericin B, which can indicate a different mechanism of action of the peptide from the drug. In our previous study, the AurH1 showed synergistic effects against *C. albicans* in addition to amphotericin B in combination with fluconazole. However, in our recent study, its synergistic effect with fluconazole was lost by the amidation of this peptide [6]. In confirmation of this hypothesis, the ergosterol binding assay showed that treating the AurH1-NH_2_ with ergosterol cannot affect its fungicidal activity. Therefore, it can be concluded that ergosterol is probably not a target for the binding of AurH1-NH_2_, which confirms the hypothesis that the AurH1-NH_2_ has a different mechanism of action from amphotericin B. On the other hand, studies of *C. albicans* surface changes under the influence of treatment with AurH1-NH_2_ show that this peptide damages the cell coat of the fungus and destroys the cell wall. Our previous studies showed that the action mechanism of AurH1 similar to AurH1-NH2 was the destruction of the fungal cell coat [6]. The combination of AurH1-NH2 and AC-AurH1 only with amphotericin B showed synergistic effects, which could be due to their different mechanism of action and non-interference of their effects against fungi. FE-SEM images of the surface morphology of *C. albicans* confirm the effect of the peptides on their cell walls. On the other hand, it was found that both peptides do not bind to ergosterol, so it can be assumed that the mechanism of action of the peptides is different from amphotericin B and they probably target the components of the cell wall. Of course, this issue needs further investigation. Therefore, it can be concluded that other wall components such as polysaccharides (Chitin, glucan, mannan, etc.) or mannoproteins are targets for AurH1 and AurH1-NH_2_. On the other hand, it is clear here that the amidation of the C-terminal of AurH1 does not play a role in changing its mechanism of action but only improves its antifungal properties.

Studies of the effect of human serum on the fungicidal potency of AurH1 and AurH1-NH2 suggest that amidation of the C-terminal of AurH1 can increase the durability of its effect against proteolytic degradation. It can be assumed that the amidated peptides become more resistant to the action of carboxypeptidases. However, these chemical modifications did not affect the salt stability of the peptides.

## Conclusion

In this study, two acylated and amidated derivatives of AurH1 antifungal peptides (Ac-AurH1 and AurH1-NH_2_) were synthesized. The results showed that amidation at the C-terminal of AurH1 compared to acylation at the N-terminal of it has been able to improve the antifungal properties of this peptide while but antifungal properties of the acylated derivative of the parent peptide have also decreased. Also, the amidation of AurH1 at its C-terminal could improve its stability and even its cytotoxicity. Also, amidation of this peptide caused more powerful anticancer properties against breast cancer cell lines than the parent peptide. However, the amidation of the C-terminal of AurH1 could not affect the mechanism of action and its time -of killing. AurH1-NH_2_, like AurH1, influences the cell wall of the fungus, but they cannot bind to ergosterol. In another conclusion, it can be said that in addition to decreasing its fungicidal potency, acylation of AurH1 increased its cytotoxicity. Also, the acylation of this peptide has not been able to affect its binding to ergosterol. In general, it can be concluded that the amidation of the C-terminal of the AurH1 is a suitable strategy to improve its antifungal properties and cytotoxicity without changing its amino acid sequence, which can strengthen its properties for animal studies.

## Data Availability

All data and materials are available in the article. Other data will be provided upon request.
